# From the Biology of PP2A to the PADs for Therapy of Hematologic Malignancies

**DOI:** 10.3389/fonc.2015.00021

**Published:** 2015-02-16

**Authors:** Maria Ciccone, George A. Calin, Danilo Perrotti

**Affiliations:** ^1^Department of Experimental Therapeutics, MD Anderson Cancer Center, The University of Texas, Houston, TX, USA; ^2^Department of Medicine, The Greenebaum Cancer Center, University of Maryland School of Medicine, Baltimore, MD, USA

**Keywords:** PP2A, PADs, SET, phosphatases, tumor suppressor

## Abstract

Over the past decades, an emerging role of phosphatases in the pathogenesis of hematologic malignancies and solid tumors has been established. The tumor-suppressor protein phosphatase 2A (PP2A) belongs to the serine–threonine phosphatases family and accounts for the majority of serine–threonine phosphatase activity in eukaryotic cells. Numerous studies have shown that inhibition of PP2A expression and/or function may contribute to leukemogenesis in several hematological malignancies. Likewise, overexpression or aberrant expression of physiologic PP2A inhibitory molecules (e.g., SET and its associated SETBP1 and CIP2A) may turn off PP2A function and participate to leukemic progression. The discovery of PP2A as tumor suppressor has prompted the evaluation of the safety and the efficacy of new compounds, which can restore PP2A activity in leukemic cells. Although further studies are needed to better understand how PP2A acts in the intricate phosphatases/kinases cancer network, the results reviewed herein strongly support the development on new PP2A-activating drugs and the immediate introduction of those available into clinical protocols for leukemia patients refractory or resistant to current available therapies.

## Introduction

The phosphorylation status of proteins is crucial in regulating their functions and properties. In normal cells, kinases and phosphatases participate to phosphorylation processes (1). By modulating the phosphorylated load, they control the on–off switch of target proteins. The balance between kinase and phosphatase activities is critical in many hematological malignancies. Although numerous studies highlighted the role of kinases in malignant transformation, much less is known about the contribution of specific phosphatases to cancer development.

Oncogenic kinases and tumor-suppressor phosphatases maintain the cell homeostasis by exerting their activities on cell growth, survival, and differentiation. Thus, aberrant oncogene expression and loss of tumor-suppressor gene function affect cell cycle, apoptosis, and DNA damage repair machineries. *ABL1* is a well-characterized kinase that has been involved in leukemic transformation (2). The break cluster region (BCR)–ABL1 oncoprotein is a tyrosine-kinase that constitutively activates downstream signaling thereby inducing cell proliferation, survival, and clonal expansion in chronic myeloid leukemia (CML) and a cohort of acute lymphoblastic leukemia (ALL) patients. In addition to the tumor-suppressors *TP53*, *RB1*, and *ATM*, usually lost in several leukemias, inhibition of specific phosphatases expression or loss of their function has been recently detected in many cancers (3). Interestingly, there is increasing evidence that unrestrained oncogene kinase activity is not sufficient to transform cells if a concurrent inhibition of the antagonizing tumor-suppressor phosphatases does not occur (4).

The introduction of tyrosine-kinase inhibitors [TKIs; e.g., imatinib (IM)] has dramatically changed the clinical outcome of patients with hematological malignancies (5). However, the occurrence of mutations at the active kinase site or additional chromosomal/molecular abnormalities resulting in drug resistance and, possibly, disease progression (6) indicate that new strategies are needed to prolong survival of leukemia patients resistant or refractory to current chemo and TKI-based therapies. Although the rescue of tumor-suppressor function seems to be an intriguing challenge in cancer treatment, few molecules have been proven to target specifically tumor-suppressors.

Protein phosphatase 2A (PP2A) refers to a family of serine–threonine phosphatases that accounts for the majority of serine–threonine phosphatase activity in eukaryotic cells (7). In the last decade, an emerging role of PP2A in the onset and progression of solid tumors and hematological malignancies has been established (8, 9). These findings led to the discovery and development of a new family of compounds, the PP2A-activating drugs (PADs) that are capable of restoring PP2A phosphatases activity of most of them through interference between PP2A and its inhibitor SET/I2PP2A (8, 10). Pre-clinical studies proved the efficacy and no-toxicity of PADs in several *ex vivo* and in animal studies (8), indicating that PP2A reactivation in combination with kinase inhibition or chemotherapy represents a feasible and effective strategy for the treatment of hematological malignancies.

## PP2A in Normal Cells

### PP2A structure and regulation

The PP2A core enzyme consists of a 36 kDa catalytic C subunit (PP2A_C_) and a 65 kDa structural A subunit (PP2A_A_). In mammals, two isoforms (α and β) are encoded for each subunit (Aα, Aβ, Cα, and Cβ) (11). The structure of the PP2A complex is enriched with a regulatory B subunit. Four families of the B subunit, with a molecular weight ranging from 50 to 130 kDa, have been identified: PP2A_B_ (B55 or PR55), PP2A_B′_ (B56 or PR61), PP2A_B″_ (B72 or PR72; B130 or PR130; PR48; and G5PR) PP2A_B-_ (PR93 and PR110). By assembling differently the A, B, and C subunit isoforms, more than 75 distinct PP2A holoenzymes could be built up (12).

The A and C subunits are evolutionary conserved and ubiquitously expressed. These two subunits form a catalytic complex (PP2A/A_C_) that can interact with the regulatory B subunits or certain viral antigens (e.g., polyoma small T and middle T antigens, and SV40 small tumor antigen) to affect activity and determine PP2A substrate and tissue specificity (12, 13). Moreover, the B subunit may vary depending on stage-development (14, 15) and it can recruit PPA2/A_C_ to a selective subcellular compartment (cytoplasm or nucleus) driving it to a specific substrate target (1).

The ability of PP2A to regulate different cellular activities depending on its structure and composition renders this enzyme a suitable target for new therapeutic compounds as improving the drug affinity for each isoform might entail high tissue specificity and better safety profile.

### PP2A pathways

**Figure 1:**
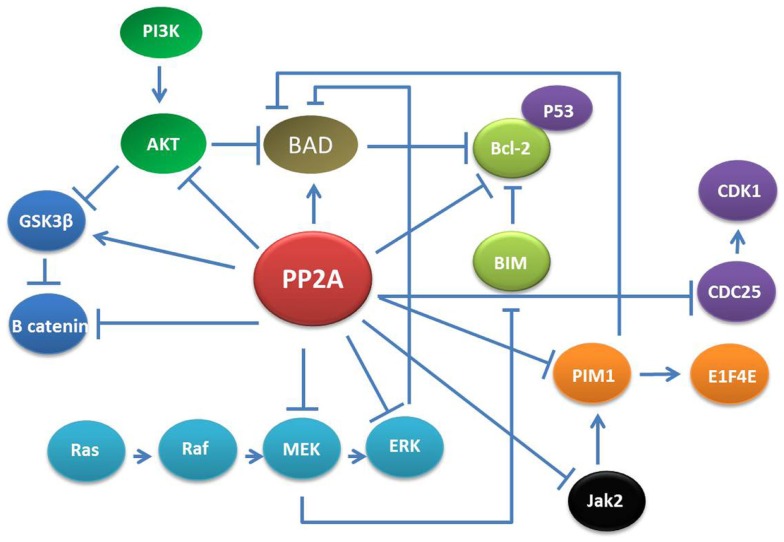
**PP2A networks**. PP2A is involved in various cellular signaling including MAPK/ERK, PI3K/AKT, Jak2, and CDK. Thus, the effects of PP2A down-regulation could potentially affect multiple pathways resulting in alteration of apoptosis, cell growth, proliferation, and differentiation in adult cells.

Protein phosphatase 2A is involved in a consistent number of physiological processes in adult and embryonic cells (Figure 1). For example, the transcription factor β-catenin, which is the major effector in the Wnt signaling pathway both in cancer and embryonic development (12, 16) undergoes PP2A-dependent regulation. PP2A also plays an essential role in cell division and apoptosis depending on the specific isoform constituting the PP2A holoenzyme (17, 18). Indeed, PP2A is required to maintain G1/S cyclin levels by modulating their phosphorylation status, a necessary event to properly transit through the cell cycle (19).

Notably, PP2A loss-of-function results in inhibition of apoptosis. This mostly depends on interference with two major mitogenic/survival pathways: the MAPK/ERK and the PI3K/AKT cascades. In normal cells, specific PP2A complexes desphosphorylate and inactivate MEK and ERK1 kinases thereby keeping the activity of many signal transducers, apoptosis regulators, and transcription factors (e.g., STAT5 and 3; c-Myc) tightly regulated. PIM1 kinase protein is a target of PP2A and once it is dephosphorylated by PP2A, it is degraded by the proteasome machinery (20). Furthermore, PIM1 represses the pro-apoptotic molecule BAD through its phosphorylation at Ser112 gatekeeper site (21). Likewise, PP2A suppresses PI3K/AKT-generated signals mostly by direct AKT dephosphorylation but also through activation of GSK-3b or inhibition of cytokine-generated signals, which lead to Akt-activation (22, 23).

In addition to the MAPK/ERK and PI3K/AKT cascades, PP2A function is strictly related with the Janus kinase 2 (Jak2) tyrosine kinase (24, 25). In IL-3-stimulated myeloid progenitor cells, the interaction between Jak2 and PP2A transiently increases and Jak2 becomes activated and PP2Ac activity suppressed by phosphorylation on Y307 (26). In primary myeloid stem and progenitors cells, reactivation of PP2A also induces Jak2 inactivation thus resulting in shut-down of mitogenic/survival cytokine-generated signals (27).

### PP2A inhibitors

Physiologic PP2A inhibitors seem to be involved in the mechanisms of progression and aggressiveness of hematological malignancies. ANP32A (I1PP2A), SET (I2PP2A), CIP2A, SETBP1A, type 2A-interacting protein (TIP), and ENSA are well-characterized cellular PP2A inhibitory molecules (Table 1).

**Table 1:** **PP2A inhibitors in hematological malignancies**.

PP2A inhibitors	Pattern of expression	Hematological malignancies
SET (I2PP2A)	Overexpression/gain of function	CML, CLL, NHL cell line, Jak2^V617^MPN, AML
SETBP1	Mutations Overexpression/gain of function	aCML, sAML, CMML, CNL, unclassified MDS/MPN
CIP2A	Overexpression	CML
ANP32A (I1PP2A)	Overexpression	K562 cell line
TIP	Unknown	Unknown
ENSA	Unknown	Unknown

*CML, chronic myeloid leukemia; CLL, chronic lymphocytic leukemia; NHL, non-Hodgkin lymphoma; Jak2 ^V617^MPN, Jak2V617F positive myeloproliferative neoplasia; AML, acute myeloid leukemia; aCML, atypical chronic myeloid leukemia; sAML, secondary acute myeloid leukemia; CMML, chronic myelomonocytic leukemia; CNL, chronic neutrophilic leukemia; unclassified MDS/MPN, unclassified myelodisplastic/myeloproliferative neoplasia*.

SET and SET binding protein (SETBP1) act synergistically and permit the formation of the SETBP1/SET/PP2A complex, which inhibits PP2A phosphatase activity (28). *SET (or I2PP2A)* is a potent endogenous inhibitor of the tumor-suppressor PP2A (8) as PP2A acts as a negative regulator of several survival and proliferation pathways that are aberrantly activated in hematological malignancies (8). In addition, it has been reported that SET inhibits the DNase activity of the tumor-suppressor NM23-H1 and that the cleavage of SET by Granzyme A during the cytotoxic T lymphocyte-induced apoptosis releases NM23-H1 from inhibition and triggers NM23-H1 to translocate into the nucleus, where it cleaves the DNA (29, 30).

#### SETBP1

*SETBP1* localizes on chromosome 18q21.1 and codes for a nuclear protein of 170-kDa that specifically interacts with SET forming a heterodimer (31). SETBP1 germline mutations have been described in Schinzel–Giedion syndrome, a rare congenital disorder characterized by aberrant bone formation (32) and predisposition to myeloid malignancies. *SETBP1* overexpression protects SET from protease cleavage, increasing the amount of full-length SET protein and leading to the formation of the SETBP1–SET–PP2A complex that results in PP2A inhibition (28). Interestingly, not only *SETBP1* overexpression but also *SETBP1* mutations have been associated with hematological disorders (28, 33, 34). Cells expressing SETBP1 Gly870Ser variant showed higher level of SETBP1 and SET proteins, and significantly reduced PP2A activity indicating that *SETBP1* mutations contribute to SET stabilization and PP2A inhibition (34).

An additional PP2A inhibitor is the *cancerous inhibitor of PP2A*, e.g., *CIP2A*, which normally prevents PP2A-mediated dephosphorylation of c-Myc at serine 62. pS^62^-Myc is more stable than its dephosphorylated counterpart, and CIP2A therefore prevents c-Myc degradation (35). Moreover, impaired p53 activity was demonstrated to increase CIP2A expression trough E2F1, which in turn, by inhibiting PP2A activity, increases stabilizing E2F1 (36).

Whereas *in vivo* data have confirmed the involvement of SET, SETBP1, and CIP2A proteins in leukemic transformation process, so far we could not rule out the contribution to the development of hematological malignancies of other molecules that have been proven to inhibit PP2A including *TIP*, *ANP32A*, and *ENSA*. Recently, one study has reported that knock-down of the NM23-H1 factor in CML K562 cell line decreased proliferation and altered the expression of ANP32A, a potent and selective PP2A inhibitor (37).

Although variations exist between increased levels of PP2A inhibitors and adverse clinical outcome in different hematologic malignancies, pharmacologic suppression of PP2A inhibitors represents an effective strategy to induce apoptosis of leukemic cells and/or sensitize resistant cells to TKIs (25, 27, 38–40). Thus, PP2A reactivation should be taken as a possible therapeutic approach.

## PP2A and Hematological Malignancies

### Chronic myeloid leukemia and myeloproliferative disorders

#### Chronic myeloid leukemia

Chronic myeloid leukemia is a myeloproliferative disorder arising from the transformation of the hematopoietic stem cell (HSC) and characterized by exuberant cell expansion and altered differentiation of myeloid precursors. The molecular hallmark of CML is the Philadelphia chromosome (Ph) that derives from the translocation t(9;22) and causes the juxtaposition of the *ABL1* kinase gene with the break cluster region gene (*BCR*) (2). The BCR–ABL1 fusion oncoprotein is a constitutively active tyrosine kinase that confers growth factor-independent proliferation and enhanced survival to the hematopoietic progenitors harboring this abnormality. Although TKIs control the disease in vast majority of patients, a small proportion of patients with CML will eventually lose response to TKIs treatment and progress to blast crisis (BC) (41). The occurrence of mutations at the active site of *BCR*–*ABL1* may at least in part explain the overcoming resistance in patients treated with TKIs (42). Moreover, the persistence of quiescent CML HSCs might contribute to disease progression because TKIs seem to not affect leukemic stem cell proliferation and survival (43). In CML cells, PP2A is a key target in BCR–ABL1 downstream signaling and, in fact, the BCR–ABL1 oncoprotein prevents PP2A auto-dephosphorylation at tyrosine 307 (44, 45) thereby keeping PP2A in its inactive status. By targeting BCR/ABL1, TKIs inhibit ABL kinase activity and restore PP2A function. Interestingly, in IM-resistant CML cells, the sphingosine kinase-1 (SK-1)/sphingosine 1-phosphate (S1P) activation enhances BCR–ABL1 stability through S1P receptor 2 (S1P2) signaling, which prevents BCR–ABL1 dephosphorylation and degradation through inhibition of PP2A (45). Molecular and pharmacological interference with SK-1/S1P2 may restore PP2A-dependent dephosphorylation and enhance IM- or nilotinib-induced growth inhibition in primary CD34^+^ CML progenitors and BCR–ABL1+ cell lines.

**Figure 2:**
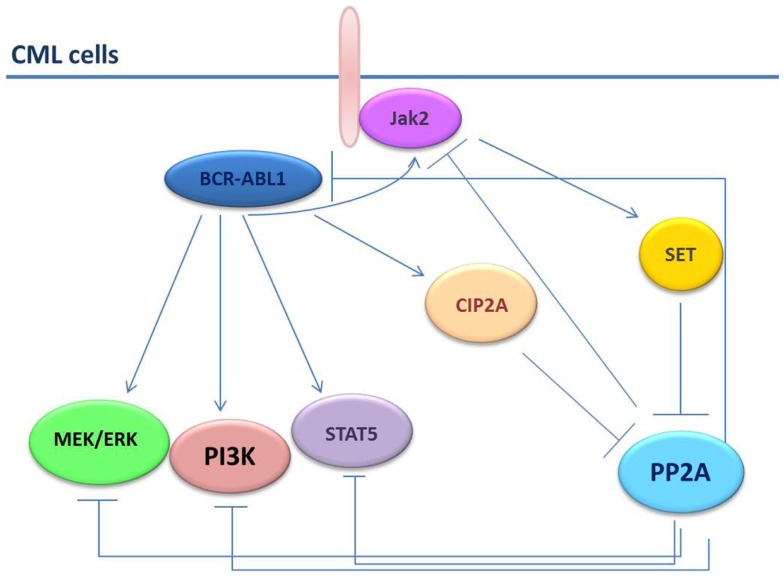
**PP2A in CML cells**. In CML cells, the BCR–ABL1 and the PP2A pathways are strictly connected. PP2A induces dephosphorylation/inactivation of BCR–ABL1 and Jak2 tyrosine kinases. Conversely, BCR–ABL1 induces SET and CIP2A expression thus increasing the inhibition on PP2A. Jak2 also down-regulates PP2A through SET.

Recently, it has been shown that persistence of leukemic HSCs in bone marrow of patients with CML requires the inhibition of the PP2A and BCR–ABL1 expression but not its activity (27). In comparison with HSCs from healthy individuals, PP2A activity was remarkable suppressed in HSCs from patients with CML (27). This result is apparently in contrast with the low BCR–ABL1 tyrosine-kinase activity found in the Ph+ HSC-enriched cell fractions, which would have expected to be associated with PP2A restoration. However, BCR–ABL1 expression is needed for the recruitment and activation of Jak2 that, in turn, triggers SET-dependent PP2A inhibition and activation of β-catenin-mediated self-renewal/survival signals (27). The consistent difference in PP2A activity in normal and leukemic HSCs implies that restoration of PP2A activity would allow the selective targeting of leukemic HSCs without compromising the normal counterpart (Figure 2).

An additional finding is that in CML stem cells not only PP2A but also PP2A inhibitory molecules might be aberrantly expressed and might contribute to low PP2A activity. *SET* was the first PP2A inhibitory molecule to have been found up-regulated in CML (CP and BC) and Ph+ B-ALL CD34+ progenitors through a BCR–ABL1 dose- and kinase-dependent manner (38). In CD34+ primary cells from patients with CML-BC and in BCR–ABL1+ cell lines, SET expression is enhanced by BCR/ABL and increases during CML disease progression. In fact, SET protein levels correlate with BCR/ABL activity and are higher in CML-BC CD34+ than CML-CP CD34+ cells and in CML-CP CD34^+^ cells than in CD34+ cells from healthy donors (44). SET-mediated PP2A inactivation is essential for survival/self-renewal of quiescent CML HSCs and maintenance of an active Jak2-β-catenin pathway and that PP2A is suppressed in CML HSCs in a BCR/ABL kinase-independent, Jak2-dependent manner (27). In BCR/ABL transformed cells, the levels of PP2A structural subunit PR65/A were found to correlate with increased levels of PP2Ac phosphorylation on tyrosine 307 which, as reported, inhibits PP2A activity (44). Accordingly, PP2ATyr307 levels are markedly reduced upon SET down regulation by means of short-hairpin RNA (shRNA) or IM treatment (44). The restoration of PP2A phosphatase activity induces the SHP-1-dependent BCR–ABL1 dephosphorylation/inactivation and triggers BCR–ABL1 proteasome degradation (44).

Likewise, CIP2A have been found overexpressed in hematological malignancies and in solid tumors, and to be associated with high proliferation rate and poor prognosis (46, 47). In a retrospective study, Lucas et al. have shown that high CIP2A protein levels are elevated at diagnosis in mononuclear cells (MNCs) and CD34+ cells from patients with CML who progressed into BC compared to patients who achieved a complete cytogenetic response (CCyR) or who had not progressed (48). Interestingly, in patients who progressed into CML-BC, the CIP2A levels were sustained despite IM treatment and low Sokal score. The inhibition of CIP2A resulted in a reduction of inactivated phosphorylated PP2ATyr307 fraction and BCR/ABL1 kinase activity. The knock-down of CIP2A led to a consistent drop of SET and Jak2 protein levels, suggesting the existence of an interplay between the PP2A complexes, their physiologic inhibitors (e.g., SET and CIP2A), and the BCR–ABL1/JAK2 signalosome (48).

Protein phosphatase 2A is also inactivated in *polycythemia vera* (PV), *essential thrombocytosis* (ET), and *primary myelofibrosis* (PMF) and other *myeloproliferative disorders* (*MPD*s) characterized by the expression of the transforming Jak2^V617F^ oncogene (25). Consistent with significant reduction of PP2A activity in CD34+ progenitors from patients with Jak2^V617F^ positive ET, PV, and MFI, treatment with Jak inhibitors may rescue PP2A activity through SET silencing (25).

Taken together, these data indicate that the balance among PP2A, SET, and CIP2A may play a crucial role not only for maintenance of CML progenitors both in chronic and blastic phase but also for the population of leukemic stem cells showing innate resistance to TKI. This observation has strong critical clinical implications as drugs that are able to rescue PP2A activity could potentially eradicate the leukemic HSCs reservoir or be effective in patients who progress to BC that so far define a clinical stage characterized by treatment refractoriness and poor prognosis.

### Myelodysplastic syndrome and myelodysplastic/myeloproliferative neoplasms

In other myeloproliferative malignancies, inhibition of PP2A is achieved by SET stabilization through its interaction with SETBP1 (31). Recently, by exome sequencing technology, *SETBP1* mutations were found to recur frequently in patients with *atypical chronic myeloid leukemia (aCML)*, a disorder with overlapping clinical features with CML but without the typical Ph1 translocation (34). Out of 644 patient samples with different myeloid and lymphoid malignancies and 344 cell hematopoietic and non-hematopoietic lines, the pGly870Ser SETBP1 mutation occurred in 24% of patients with aCML, and in 25, 10, and 4% of patients with chronic neutrophilic leukemia (*CNL)*, unclassified MDS/MPN disorders, and chronic myelomonocytic leukemia (*CMML*), respectively. Ectopic SETBP1 Gly870Ser expression in TF1 enhanced cell proliferation, which likely resulted from suppression of SETBP1 ubiquitination/degradation that, in turn, led to SET stabilization and PP2A inhibition (34). In primary aCML cells as well as in cells overexpressing the mutated SETBP1, SETBP1 levels correlated with increased expression of *Lyn* and *PTGS2* (two known targets of PP2A) and induction of the TGF-β pathway. Interestingly, aCML patients with *SETBP1* mutations had a worse prognosis than those with wild-type *SETBP1* (34).

Similar to what was found in aCML, a different group identified recurrent somatic mutations in *SETBP1* by whole-exome sequencing of individuals with various myeloid malignancies (49). The mutations (pAsp868Asn, pGly870Ser, pIle871Thr, pSer869Asn, and pAsp880Asn or pAsp880Glu) in this cohort of patients corresponded with the recurrent *de novo* germline mutations responsible of Schinzel–Giedion syndrome and were detected in 15% of CMML cases and 17% of secondary acute leukemia (sAML). Mutant cases were associated with advanced age, poor cytogenetic abnormalities, and shorter survival. However, in CMML subgroup, *SETBP1* retained its prognostic value in association with *CBL* mutations. Furthermore, the sequential analysis of serial samples at multiple time points of the disease course, in two different series of patients with myeloid malignancies, showed that *SETBP1* mutations were absent at the time of initial presentation while increasing in allele frequencies by time (49, 50). These observations suggest that *SETBP1* mutations are acquired during leukemic evolution contributing to leukemic progression rather than leukemic transformation. Transduction with mutant *SETBP1* led to the immortalization of mouse myeloid precursors that acquired enhanced proliferative capacity and tyrosine phosphorylation of PP2A. In addition, a significantly higher *HOXA9* and *HOXA10* expression levels were observed in SETBP1-mutant leukemias in comparison with wild-type cases without *SETBP1* overexpression (49). Overall, *SETBP1* mutations predict poor outcome irrespective of age, sex, International Prognostic Scoring System (IPSS), and unfavorable cytogenetic risk (49, 50). However, the adverse prognostic value of *SETBP1* mutations was not confirmed in two cohorts of patients with aCML and CMML, probably due to the fact that the median follow-up was relatively short (33).

Furthermore, SETBP1-mutant cases have been associated with higher white blood cell counts, high grade thrombocytopenia, and anemia than wild-type cases (33), with poor risk cytogenetic abnormalities like monosomy 7/del7q (33, 49, 50) and isochromosome i(17) (33, 50). Interestingly, in patients with i(17)(q10), the *TP53* and *SETBP1* mutations are mutually exclusive; likewise, *SETBP1* mutations rarely occur in cases harboring Jak2^V617F^, MPL^W515^, Jak2 exon 12, and *TET2* mutations (33). Conversely, *ASXL1* and CBL mutations correlate significantly with the occurrence of *SETBP1* mutations (33, 34, 49, 50).

Although further are warranted, *SETBP1* mutations seem to define a subgroup of patients with myeloid disorders characterized by specific clinical features and worse prognosis.

### Acute myeloid leukemia

An increasing number of studies have shown that the PP2A pathway is also affected not only in CML but also in other myeloid malignancies. Over the past few years, large-scale genomic studies have unveiled the presence of novel genetic aberrations in patients with *acute myeloid leukemia (AML)*. C-KIT is a type-3 tyrosine-kinase receptor activating multiple proliferation, differentiation, and survival signals in stem cells (51). Activating c-KIT mutations have been differently associated with higher relapse rate, shorter survival, and TKIs resistance (52). Interestingly, PP2A structural and regulatory subunits were found significantly reduced in myeloid cells expressing activated c-KIT mutants, meaning that PP2A is a crucial mediator of c-KIT leukemogenesis (40). Accordingly, the overexpression of PP2A-Aα in D816V c-KIT cells induced apoptosis and inhibited proliferation (40), suggesting that the restoration of PP2A activity could represent an effective strategy to overcome drug resistance in c-KIT^+^ AML.

Cristobal et al. described a novel translocation t(12;18)(p13;q12) involving ETV6 in a patient with AML. The translocation resulted in overexpression of SETBP1 (18q12), located close to the breakpoint. They demonstrated that in cell lines transfected with a plasmid expressing *SETBP1*, its ectopic expression leads to increased full-length SET protein levels. In fact, the overexpression of *SETBP1* might protect SET from protease cleavage and promotes the formation of a SETBP1/SET/PP2A complex that results in PP2A inhibition and increased proliferation rate of the leukemic cells (28). By quantitative RT-PCR, they observed that overexpression of *SETBP1* occurred in 27.6% of patients with AML and was associated with unfavorable cytogenetic prognostic group, monosomy 7, and *EVI1* overexpression. Similarly to patients with *SETBP1* mutations, overexpression of *SETBP1* in AML has also been associated with significantly shorter overall survival especially in elderly population (28). Accordingly, increased pY^307^ PP2A levels were observed in 78.4% of a cohort of patients that included *de novo* and secondary AML (53). In approximately two-third of patients, the PP2A inhibition was attributable to SET and/or SETBP1 overexpression. However, 34% of the patients with inactivated PP2A had either FLT3 or NPM1 aberrations, suggesting that PP2A is a secondary rather than a primary event in the process of leukemogenesis (53). Recently, SET expression studies in AML cell lines and CD34+ progenitors from healthy individuals and AML patients revealed that it was significantly increased in the cell lines tested as well as in primary patient samples harboring FLT3-ITD, NRAS^Q61L^, and JAK3^A572V^ genetic lesions (54).

In AML, the coexistence of PP2A-pathway abnormalities with well-known unfavorable molecular markers may provide novel explanations for the worse prognosis associated with those cases.

### Acute lymphoblastic leukemia

Almost 20% of adult patients affected by B-cell ALL harbor the Philadelphia chromosome translocation Ph1, t(9;22)(q34;q11), which usually differs from CML cases because the *BCR/ABL1* gene product has a molecular weight of 190 kDa (p190) (55). Although the inclusion of TKIs in the treatment of *Ph1^+^* *ALL* has significantly improved the proportion of patient achieving complete remission, it remains an incurable disease with a very dismal prognosis (56). In Ph1 ALL progenitors, the inhibition of PP2A activity occurs in a SET-dependent manner and the reactivation of the PP2A by FTY720 causes growth inhibition and induction of caspase-dependent apoptosis toward p190 down-regulation (38). Interestingly, the induction of apoptosis and inhibition of cytokine-dependent clonogenic potential are observed regardless of the presence of T315I mutation and without compromising normal myelopoiesis, stressing the concept that FTY720 could represent a feasible and effective treatment also in highly resistant Ph1+ ALL (and CML) cases (38).

#### T-cell ALL

*T-cell ALL (T-ALL)* is a malignancy often characterized by aggressive clinical outcome, high relapse risk, and poor prognosis (57). Recurrent chromosomal aberrations and gene mutations have been identified, although a consistent proportion of patients lack molecular or cytogenetic abnormalities (58). By combining array-CGH (comparative genomic hybridization) and gene expression profiling (GEP), a recurrent deletion at 9q34 was identified among patients with T-ALL (59). The del(9)(q34.11q34.13) results in *SET–NUP214* fusion gene that binds in the promoter regions of the HOXA gene cluster leading to transcriptional activation of *HOXA9* and *HOXA10* probably via CRM1 and DOT1L. In this study, the knock-down of *SET–NUP214* by siRNA down regulated the *HOXA* expression levels with only mild effect on cell proliferation and apoptosis. Although conceptually SET–NUP214 fusion protein might affect PP2A activity, no evidence has been revealed in this regard.

Although the paucity of data regarding the involvement of PP2A or SET in ALL could not draw any conclusive remark, the presence of the Ph1 chromosome in a cohort of patients with ALL would allow in the next future to investigate the role of PADs in this subgroup of patients and to test whether the inclusion of PADs in standard treatment schedule may add any advantage in cure rate and survivals.

### B-chronic lymphocytic leukemia and non-Hodgkin lymphomas

*B-chronic lymphocytic leukemia (B-CLL)* is the most common leukemia in the Western world, and is characterized by a highly heterogeneous clinical course (60). In a selected group of patients with CLL harboring deletion 11q-, reduced PP2A-Aβ subunit (PPP2R1B) transcript levels were observed. Likewise, alternative splicing of PP2A-Aβ transcripts was associated with a reduced activity of PP2A (61). To further confirmation that PP2A signaling is involved in lymphoid malignancies, *SET* was found overexpressed in primary CLL cells and B-cell *non-Hodgkin lymphoma (NHL)* cell line cells in comparison with normal B-cells (62). In patients with CLL, elevated SET levels associated differently with known prognostic factors, e.g., Rai stage, CD38 expression, and IGVH mutational status. Moreover, in CLL cells, increased levels of SET correlated significantly with disease severity (shorter time to treatment and overall survival) (62).

The crucial role of PP2A tumor suppressor in NHL is confirmed by the promising results that FTY720 has shown in pre-clinical experiments in *mantle cell lymphoma* (MCL), an aggressive B-cell malignancy characterized by t(11;14) translocation and cyclin D1 expression (39, 63). In MCL cell lines, the FTY720-induced cytotoxicity occurs independent of caspase activation and is associated with downmodulation of cyclin D1 (39, 63), phospho-Akt (39), and the formation of reactive oxygen species (ROS) (63) and lysosomal membrane permeabilization (39).

The demonstration that the PP2A pathway is affected either in myeloid or in lymphoid chronic and acute malignancies underscores the importance of this tumor suppressor and widens the spectrum of malignancies that may potentially be treated by PADs.

## PP2A and miRNA

MiRNAs are small (20–24 nucleotide) non-coding RNAs that target messenger RNA for degradation or translational repression and also bind RNA binding protein to control their activity on mRNAs (64–66). Similarly to coding gene, miRNAs could act as oncogene or tumor suppressor depending on how their expression levels associate with cancer (67). Although the contribution of miRNAs to the leukemic transformation or progression has been clearly demonstrated by different groups, few studies have focused on the possible interplay between PP2A pathway and miRNAs in hematological malignancies. Mavrakis and collaborators found that miR-19 is highly expressed in ALL and in aggressive B-cell lymphoma; in a single patient with T-ALL, they identified two novel translocations, t(9;14)(q34;q11) and t(13;14)(q32;q11), involving simultaneously Notch1 and the 17–92 cluster including miR-19 (68). Using an unbiased shRNA screening approach, they found eight genes whose knock-down resembles miR-19 effects *in vitro*, including BIM and PP2A (Ppp2r5e). Hence, in Notch-induced T-ALL miR-19 overexpression may repress PP2A and BIM, thus impairing cell survival and apoptosis (68, 69).

Except for miR-19, only a few data have provided evidence of the possible role of miRNAs in regulating PP2A activity and mostly relate to miRNA targeting specific PP2A B subunits (70). MiRNAs might inhibit PP2A transcription (similarly to miR-19) or regulate the expression of PP2A inhibitory molecules (e.g., CIP2A and SET) (71). Moreover, miRNAs can indirectly affect the PP2A pathway by altering the expression of many proteins that cross-talk with PP2A.

## PP2A-Activating Drugs

Genetic (SET shRNA-mediated down-regulation) or pharmacologic restoration (i.e., PADs) of PP2A activity halts malignant cell survival and proliferation both *in vitro* and in different animal models of leukemia.

The first drugs to be tested were *forskolin*, a diterpene derived from the roots of *Coleus forskohlii*, and its derivative *1,9-dideoxyforskolin*, which lacks the adenylate cyclase activity but retains the ability to inhibit PP2A (44) (Table 2). In CML and in Ph1^+^ ALL, forskolin may suppress BCR/ABL1 oncogenic potential *in vitro*. Interestingly, the anti-leukemic effects of forskolin was observed either in IM-sensitive or in -resistant BCR/ABL1^+^ cell lines including cell lines harboring the T315I mutation that confers resistance to most TKIs (44). Furthermore, PP2A-activation results in growth suppression, enhanced apoptosis, restored differentiation, impaired clonogenic potential, and decreased leukemogenesis in SCID mice injected with BCR/ABL1^+^ cells regardless of their sensitivity to IM (38, 44). Importantly, forskolin and 1,9-dideoxyforskolin do not affect CD34^+^ normal cells, thus preventing toxic effects on normal hematopoiesis (38).

**Table 2:** **PP2A-activating drugs (PADs)**.

Drugs	Mechanism of action	Phase study	Hematological malignancies
Forskolin/1,9-dideoxyforskolin	PP2A reactivation	I	CML (included T315I)
Fingolimod OSU-2S S-FTY720	SET inhibitor	I	CML, AML ALL Ph^+^ and Ph^−^ Jak2^V617^MPN B-CLL and MCL
OP449	SET inhibitor	I	B-CLL, NHL, CML, AML

*CML, chronic myeloid leukemia; ALL, acute lymphoid leukemia; Jak2V617MPN, Jak2V617F positive myeloproliferative neoplasia; B-CLL, B-cell chronic lymphocytic leukemia; NHL, non-Hodgkin lymphoma; MCL, mantle cell lymphoma; AML, acute myeloid leukemia*.

Although forskolin and 1,9-dideoxyforskolin showed good safety profile and efficacy in pre-clinical studies, the following study-drug phases focused on a new non-immunosuppressive compound, e.g., *FTY720* (Fingolimod, Gylenia™), recently approved for the treatment of multiple sclerosis and for prophylaxis of solid organ transplantation rejection (72, 73). After phosphorylation by sphingosine kinase 2 (SPHK2), FTY720-P temporary promotes internalization of the sphingosine 1-phosphate receptor (S1PR1). The downregulation of S1PR1 on the surface of lymphocytes prevents their egress from the thymus or lymph-nodes to blood stream. However, S1P/S1PR1 signaling may modulate T-cell trafficking as well as T-helper priming, probably through the sequestration of T-cells within the antigen encounter sites and the regulation of activation molecule receptors (74). The high oral bioavailability and the absence of harmful side effects prompted the experimentation of FTY720 into clinical phases.

The anti-leukemic effect of FTY720 is thought to rely on the ability to re-activate PP2A and it does not require SPHK2 phosphorylation (75). The S1PR1 agonist (FTY720-P) neither induces PP2A activity nor antagonizes the specific molecular aberrations, for example, BCR–ABL1 or Jak2 pathways (8, 25). By contrast, the FTY720 derivatives (e.g., OSU-2S and S-FTY720-regioisomer) do not bind at the docking site of S1PR1 and do not get SPHK2 phosphorylated thereby inactive as immunosuppressive drugs while they still retain the ability to bind SET and activate PP2A (25, 27, 76). Thus, this second generation FTY720 derivatives will overcome the possible increased conversion to the inactive FTY20-P by lymphoid B and T cells expressing SPHK2 thereby preventing the adverse effects (e.g., lymphopenia or bradycardia), which have been noted in patients with multiple sclerosis at the time of FTY720 therapy initiation (72).

So far, the data of FTY720 efficacy in hematological malignancies are drawn from pre-clinical studies, although the drug has been used in patients with multiple sclerosis. Interestingly, in bone marrow xenografts, PADs nearly abrogated survival of leukemic progenitors and dramatically impaired survival and self-renewal of CML and Ph1^+^ B-ALL stem and/or progenitor cells, but not of normal quiescent HSCs, through BCR–ABL1-independent and PP2A-mediated inhibition of Jak2 and β-catenin (27, 44). This results in apoptosis of CD34^+^ progenitors in patients with TKI-sensitive and -resistant CML and translates into long-term survival with normal myelopoiesis and absence of toxic effects in BCR/ABL1-positive leukemic mice (27, 38, 44). These findings could represent a model to selectively target HSCs reservoir sparing normal stem cells. The depletion of leukemic and genetically unstable stem cells has several biological and clinical implications. At first, it would prevent the onset of Ph1 negative sub-clones, which may eventually expand and contribute to disease progression or relapse in all types of Ph^+^ leukemias (77). Second, PADs seem to selectively activate PP2A in leukemic HSCs without harmful events on normal hematopoiesis; this could be the rationale to test the efficacy of PADs in other stem cell-derived malignancies. At last and most importantly, the eradication of leukemic HSCs would theoretically cure patients with CML who may eventually discontinue TKIs treatment.

Recently, Oaks and collaborators provided evidence of FTY720 efficacy in Jak2-driven hematological malignancies (25). PP2A is inactive in PV and in Jak2^V617F^ MPN progenitors, and this occurs in an oncogene dose- and kinase-dependent manner and it is mediated by PP2A inhibitor SET. In Jak2^V617F^ MPN cells, the FTY720 non-immunosuppressive derivatives (OSU-2S and S-FTY720-regioisomer) down-regulate Jak2 through the binding with SET lysine K209, which finally causes the sequestration and inactivation of SET (25). Of interest, in SCID mice injected with BA/F3-Jak2^V617F^ cells, FTY720 long-term treatment is associated with longer survival and decreased leukemic allelic burden without any injury on cardiac performance.

In myeloid and lymphoid cell lines, treatment with FTY720 restores Bcl-2 dephosphorylation and apoptosis (78, 79). However, treatment of primary CLL cell lines with FTY720-induced down-regulation of Mcl-1 but not Bcl-2 and activation of ERK1/2 dephosphorylation. These findings may be consistent with the hypothesis that FTY720 apoptosis, in CLL, is Mcl-1 and ERK1/2-mediated (80). FTY720 induces apoptosis in a time- and dose-dependent manner in primary MCL tumor cells and MCL cell lines *in vitro*. In addition, in MCL cells FTY720 treatment results in the accumulation of cells in G0–G1 and G2–M phases of the cell cycle. Most importantly, the *in vivo* therapeutic activity of FTY720 was shown in severe combined immunodeficient mice engrafted with Jeko MCL cell line (63). Interestingly, the rate of cell death is significantly improved in MCL cell lines treated with FTY720 and milatuzumab, a fully humanized monoclonal antibody (mAb) specific for CD74 that increases following induction of autophagic–lysosomal pathway by FTY720 (39).

Pharmacologic activation of PP2A by FTY720, but not FTY720-P (phosphorylated), reduced proliferation, inhibited clonogenic potential, and induced apoptosis in cells harboring the c-KIT mutation, that is frequently encountered in myeloid malignancies, especially AML (40). Similarly, *in vivo* treatment with FTY720 delayed growth of c-KIT^+^ mutant cells as confirmed by shrinkage in spleen size and significantly lower bone marrow infiltration (40).

### 

#### OP449 (also called COG449)

*OP449 (also called COG449)* is a novel, cell penetrating peptide, which acts through the binding with SET, thus preventing the formation of the SET–PP2A complexes (10). Interestingly, OP449 determined a reduction in cellular levels of the antiapoptotic Bcl-2 family member Mcl-1, which is overexpressed in CLL cells (62). In CML and AML cell lines, OP449 in combination with tyrosine kinases and chemotherapy produced efficient and selective inhibition of leukemia cell growth as compared with normal cells, suggesting that SET inhibitors and TKIs or chemotherapy could represent a new drug combination to overcome drug resistance (54). In B-CLL and NHL, OP449 has shown to increase significantly the activity of PP2A and to inhibit growth of tumor xenografts in mice (62).

#### AAL(S), (2-amino-4-(4-heptyloyphenol)-2-methylbutanol)

*AAL(S), (2-amino-4-(4-heptyloyphenol)-2-methylbutanol)* is a new non-phosphorylatable FTY720 analog that has recently shown to reduce airway hyperreactivity and inflammation in an asthma mouse model (81). In the next future, new FTY720 analogs may be tested in leukemic cells or mouse model to assess their efficacy and tolerability in hematological disorders.

Compounds that are able to target tyrosine kinases along with drugs that act at the epigenetic modifications occurring in leukemic cells are emerging as highly effective in numerous hematological malignancies (82). *Sorafenib* (a new generation FLT3 mutant inhibitors) and *vorinostat* (a histone deacetylases inhibitor, HDAC) induce tumor-cell death through the induction of PP2A activity in C16 dihydroceramide-dependent manner (83). Finally, the induction of complete differentiation of leukemic cells through the enhanced expression of PP2A regulatory subunits represents a mechanism explaining the efficacy of *methylprednisolone* in the treatment of lymphoid malignancies (84).

## Conclusion

Protein phosphatase 2A is a well-characterized phosphatase that has been found suppressed in hematological malignancies. Over the past decades, new evidence of its emerging role as tumor suppressor has been clearly provided. The overexpression or aberrant expression of PP2A inhibitory molecules, like SET or SETBP1 and CIP2A, contributes to PP2A loss-of-function. In other cases, PP2A activity is suppressed by unrestrained oncogene signaling driven by aberrant kinase activity (e.g., BCR–ABL1, Jak2^V617F^). Numerous studies have shown that PP2A loss-of-function predict shorter survival independently from other prognostic factors in myeloid and lymphoid malignancies, and disease progression/TKI response in CML. An interesting finding that has emerged recently is that suppression of PP2A is crucial for the persistence of leukemic HSCs in CML. Hence, PP2A re-activating drugs might enable to selectively target the leukemic HSCs that show innate resistance to TKIs allowing the eradication of the leukemic reservoir and the risk of disease relapse or progression. FTY720 and its non-immunosuppressive derivatives offered promising results in pre-clinical models. Although further studies are warranted to better understand how PP2A inhibition influences leukemia emergence, maintenance, and/or progression, the data reviewed herein strongly indicate that clinical trials are now imperative to evaluate the efficacy of PADs in the treatment of patients with hematologic malignancies.

## Author Contributions

Maria Ciccone, George A. Calin, and Danilo Perrotti wrote the manuscript.

## Conflict of Interest Statement

The authors declare that the research was conducted in the absence of any commercial or financial relationships that could be construed as a potential conflict of interest.
